# Methods for stroke severity assessment by chart review in the Atherosclerosis Risk in Communities study

**DOI:** 10.1038/s41598-022-16522-7

**Published:** 2022-07-19

**Authors:** Silvia Koton, Shalom Patole, Julia M. Carlson, Taylor Haight, Michelle Johansen, Andrea L. C. Schneider, James Russell Pike, Rebecca F. Gottesman, Josef Coresh

**Affiliations:** 1 Sackler Faculty of Medicine, The Herczeg Institute on Aging and The Stanley Steyer School of Health Professions, Tel Aviv University, 6997801 Tel Aviv, Israel; 2 Department of Epidemiology, Johns Hopkins University School of Public Health, Baltimore, MD USA; 3 Department of Neurology, Massachusetts General Hospital, Boston, MA USA; 4 Department of Neurology, Virginia Commonwealth University, Richmond, VA USA; 5 Department of Neurology, Johns Hopkins University School of Medicine, Baltimore, MD USA; 6 Department of Neurology, University of Pennsylvania Perelman School of Medicine, Philadelphia, PA USA; 7 Department of Biostatistics, University of North Carolina, Chapel Hill, NC USA; 8 Stroke Branch, National Institute of Neurological Disorders and Stroke Intramural Research Program, NIH, Bethesda, MD USA

**Keywords:** Neurology, Neurological disorders, Stroke

## Abstract

Stroke severity is the most important predictor of post-stroke outcome. Most longitudinal cohort studies do not include direct and validated measures of stroke severity, yet these indicators may provide valuable information about post-stroke outcomes, as well as risk factor associations. In the Atherosclerosis Risk in Communities (ARIC) study, stroke severity data were retrospectively collected, and this paper outlines the procedures used and shares them as a model for assessment of stroke severity in other large epidemiologic studies. Trained physician abstractors, who were blinded to other clinical events, reviewed hospital charts of all definite/probable stroke events occurring in ARIC. In this analysis we included 1,198 ischemic stroke events occurring from ARIC baseline (1987–1989) through December 31, 2009. Stroke severity was categorized according to the National Institutes of Health Stroke Scale (NIHSS) score and classified into 5 levels: NIHSS ≤ 5 (minor), NIHSS 6–10 (mild), NIHSS 11–15 (moderate), NIHSS 16–20 (severe), and NIHSS > 20 (very severe). We assessed interrater reliability in a subgroup of 180 stroke events, reviewed independently by the lead abstraction physician and one of the four secondary physician abstractors. Interrater correlation coefficients for continuous NIHSS score as well as percentage of absolute agreement and Cohen Kappa Statistic for NIHSS categories were presented. Determination of stroke severity by the NIHSS, based on data abstracted from hospital charts, was possible for 97% of all ischemic stroke events. Median (25%-75%) NIHSS score was 5 (2–8). The distribution of NIHSS category was NIHSS ≤ 5 = 58.3%, NIHSS 6–10 = 24.5%, NIHSS 11–15 = 8.9%, NIHSS 16–20 = 4.7%, NIHSS > 20 = 3.6%. Overall agreement in the classification of severity by NIHSS category was present in 145/180 events (80.56%). Cohen’s simple Kappa statistic (95% CI) was 0.64 (0.55–0.74) and weighted Kappa was 0.79 (0.72–0.86). Mean (SD) NIHSS score was 5.84 (5.88), with a median score of 4 and range 0–31 for the lead reviewer (rater 1) and mean (SD) 6.16 (6.10), median 4.5 and range 0–36 in the second independent assessment (rater 2). There was a very high correlation between the scores reported in both assessments (Pearson r = 0.90). Based on our findings, we conclude that hospital chart-based retrospective assessment of stroke severity using the NIHSS is feasible and reliable.

Stroke severity is the most important predictor of post-stroke outcome^1^ and has consistently been associated with short- and long-term mortality and disability after stroke^2–6^.

In the Atherosclerosis Risk in Communities (ARIC) prospective study, broad information on personal characteristics, socioeconomic data and prevalence of cardiovascular risk factors was collected from 15,792 Black and White individuals through interviews and physical exams at baseline, and follow-up exams, phone interviews, and active surveillance of discharges from local hospitals^7^. Although ARIC includes high quality individual-level information on strokes in a large cohort collected starting in 1987–1989; direct measures of stroke severity on admission had not previously been collected. The National Institutes of Health Stroke Scale (NIHSS) is a simple, valid, reliable, and widely used systematic tool for quantitative measurement of ischemic stroke‐related neurological deficits^8^. Moreover, regulators require adjustment of stroke outcomes for stroke severity, and the NIHSS has become the generally accepted and agreed metric for regulatory compliance^9^; therefore adding data on NIHSS for stroke events occurring in ARIC is important.

In 2018, we initiated a nested study aimed at collecting data from ARIC stroke de-identified hospital charts. In this manuscript, our goals are to demonstrate the feasibility of generating retrospective NIHSS ratings in a large epidemiologic study, to show that chart-based NIHSS ratings are reliable, and to document the procedures in ARIC and share them as a model for assessment of stroke severity in other large epidemiologic studies.

## Methods

### Study setting

The Atherosclerosis Risk in Communities (ARIC) study is a community-based prospective cohort study, including 15,792 adults 45–64 years old at baseline (1987–1989). Participants were sampled from four US communities: Forsyth County, North Carolina; suburbs of Minneapolis, Minnesota; Washington County, Maryland; and Jackson, Mississippi^7^.

In ARIC, stroke events and deaths are identified since 1987–1989 through review of hospital records as well as phone interviews initially conducted annually, then semi-annually from 2012, and in-person ARIC visits^10^. As has been standard practice in ARIC since the initiation of the cohort, after an initial computer-generated algorithm is applied^11^, stroke physician reviewers verify stroke events and adjudicate those verified events as definite, probable, or possible ischemic or hemorrhagic strokes. In cases of disagreement with the algorithm, a second physician review is conducted^11^. However, data on stroke severity had not been collected.

In order to determine severity of stroke events, in 2018, we initiated a nested study to collect data from ARIC stroke de-identified hospital charts that were previously adjudicated as definite or probable incident or recurrent stroke events, as part of ongoing surveillance in ARIC. All ischemic stroke ARIC charts were reviewed and data on all items in the NIHSS were evaluated according to an algorithm for retrospective collection of the NIHSS score that has been shown to be valid across the entire stroke severity spectrum^12,13^. For the present analysis, we included events occurring in ARIC from 1987–1989 to December 31st, 2009 that were adjudicated as definite or probable ischemic strokes. Standardized criteria for ischemic stroke have previously been reported^11^. Data on severity of stroke were collected and managed using the secure, web-based software platform REDCap (Research Electronic Data Capture) electronic data capture tools hosted at Johns Hopkins University^14,15^. The data that support the findings of this study are available from the corresponding author upon reasonable request.

### Chart-based retrospective assessment of NIHSS

Before initiation of this study, participating reviewers, who were all physicians, were required to complete the NIH Stroke Scale online training (https://learn.heart.org/lms/nihss) and obtain a certificate of completion issued by the American Stroke Association. Following certification, they all participated in a training session aimed at achieving a standardized approach for retrospective assessment of NIHSS using data in ARIC stroke charts. Training included cross-review of 20 charts of incident/recurrent stroke events randomly selected.

Collection of data from charts of all ischemic stroke events in ARIC from 1987–1989 to December 31st, 2009 was conducted by a lead abstraction physician to minimize inter-rater variation. The following information available in hospitalization charts was used to evaluate severity of stroke on admission: admission notes and discharge summary, imaging reports (magnetic resonance imaging scans, computerized tomography scans, carotid ultrasound, angiographies and other imaging reports if available), neurology consult notes, progress notes, clinical evaluation and examination of the patient and autopsy reports if available. We aimed to assess stroke severity on admission; therefore, we prioritized abstraction of information from neurological evaluations reported in notes on admission. If there was more than one neurological evaluation in the admission notes, we used information reported for the first exam. In case of inconsistencies in the available data, we looked for additional evidence. Our priorities for collection of data were: 1. Neurology consult (if performed on admission or soon after admission), 2. Admission notes, 3. Other notes. According to the algorithm for retrospective evaluation of NIHSS, missing physical examination data were scored as normal^13^. This approach was suggested by Williams et al. based on the assumption that neurological examination items not mentioned in the hospitalization charts are usually normal^13^. In cases in which there was doubt regarding the determination of severity level, all the existing data were re-examined in a process led by the project PI (SK) and an agreement on the severity score was reached.

Because of variability of available records, and the wide range of time from which these records were available, each reviewer reported both the NIHSS estimated score and the estimated severity category, classified into 5 levels according to the NIHSS score: NIHSS ≤ 5 (minor), NIHSS 6–10 (mild), NIHSS 11–15 (moderate), NIHSS 16–20 (severe), and NIHSS > 20 (very severe). Completeness and quality of data were routinely assessed during the entire process of data review and abstraction by the project PI (SK), in order to assure collection of valid information.

### Reliability assessment

For the assessment of inter-rater reliability, a second review was conducted, independently from the first review by the lead reviewer, by one of the four additional physician reviewers that went through the training process. Overall, 180 charts (15% of all definite/probable ischemic strokes) were reviewed by a second reviewer.

Interrater correlation coefficients for continuous NIHSS score and percentage of absolute agreement and Cohen Kappa Statistic for NIHSS categories were calculated. Symmetry test was performed to evaluate possible differences by raters in the propensity to select higher and lower NIHSS categories. We did not assess intra-rater reliability since we felt that, if given the same case to review twice, the probability a reviewer would remember their previous classification of severity would be high, due to the comprehensive review of all the documents in the stroke charts conducted in order to abstract severity data.

### Ethics approval and consent to participate

The ARIC study protocol was approved by the Institutional Review Board (IRB) of each participating center, and informed consent was obtained from participants at each study visit. The present study was reviewed and approved by the Johns Hopkins University IRB. The informed consent included permission to obtain hospital records and research was performed in accordance with relevant guidelines and regulations.

## Results

After the training period, chart review and abstraction of data required on average 15 min per chart, with a range of 10–35 min, depending on the extent of data available in the charts.

Among 1159 (96.7%) events with information required to define the NIHSS total score, median (25–75%) NIHSS score was 5 (2–8). Definition of the exact NIHSS total score was not possible for 39 events; however, in 3 out of these 39 events, charts included information that allowed to define severity by NIHSS category. Therefore, severity group was determined for 1162 (97%) events. The distribution of NIHSS categories was NIHSS ≤ 5 = 58.3% (n = 677), NIHSS 6–10 = 24.5% (n = 285), NIHSS 11–15 = 8.9% (n = 103), NIHSS 16–20 = 4.7% (n = 55), NIHSS > 20 = 3.6% (n = 42). To note, determination of severity category was not possible in 36 ischemic stroke events due to absence of relevant notes or very scarce information in the charts.

The participant characteristics associated with stroke events included in the interrater agreement sample were similar to those excluded from the interrater agreement sample with the exception of diabetes (21.9% versus 31.5%, *p* = 0.01) (Table 1). Mean age at stroke was higher in the interrater agreement sample (72.9 years versus 68.0 years, *p* < 0.001).

**Table 1:** Baseline characteristics of ARIC participants with ischemic stroke, overall and by inclusion in the inter-rater reliability assessment.

	Ischemic Stroke Events	Included in Reliability Sample	*Not* Included in Reliability Sample	*p*-value
Sample size, N	1198	180	1018	
Age at stroke, mean (SD), years	68.8 (7.6)	72.9 (5.9)	68.0 (7.6)	< .001
Age at baseline (1987–1989), mean (SD), years	56.3 (5.6)	55.9 (5.2)	56.2 (5.6)	0.38
Female sex, No. (%)	503 (49.4)	99 (55.0)	602 (50.3)	0.17
**Race and center, No. (%)**				0.20
White, Forsyth County, North Carolina	193 (16.1)	37 (20.8)	156 (15.3)	
Black, Forsyth County, North Carolina	55 (4.6)	3 (1.7)	52 (5.1)	
White, Minneapolis, Minnesota	215 (18.0)	32 (18.0)	183 (18.0)	
White, Washington County, Maryland	275 (23.0)	17 (9.6)	258 (25.3)	
Black, Jackson, Mississippi	454 (38.0)	89 (50.0)	365 (35.9)	
**Education, No. (%)**				0.84
< High school	448 (37.4)	63 (35.0)	385 (37.9)	
High school, GED, or vocational school	421 (35.2)	74 (41.1)	347 (34.1)	
College, graduate, or professional school	328 (27.4)	43 (23.9)	285 (28.0)	
**Smoking status, No. (%)**				0.06
Current	391 (32.7)	52 (28.9)	339 (33.3)	
Former	348 (29.1)	46 (25.6)	302 (29.7)	
Never	458 (38.3)	82 (45.6)	376 (37.0)	
Body mass index	29.0 (5.5)	28.6 (5.4)	29.0 (5.6)	0.34
Use of hypertension medication	583 (48.7)	79 (43.9)	504 (49.5)	0.16
Diabetes	354 (30.1)	39 (21.9)	315 (31.5)	0.01
Use of statins	7 (0.6)	2 (1.1)	5 (0.5)	0.32

The distributions of NIHSS categories by rater, percent agreement and Cohen’s Kappa statistic are displayed in Table 2. Overall agreement in the classification of severity by NIHSS category was present in 145/180 events (80.56%). Symmetry test was non-significant (*p* = 0.79) suggesting that the two raters who were reviewing the same chart had the same propensity to select higher and lower NIHSS categories. Cohen’s simple Kappa statistic (95% CI) was 0.64 (0.55–0.74) and weighted Kappa was 0.79 (0.72–0.86).

**Table 2:** Distribution of NIHSS category by rater and inter-rater agreement measures, N = 180.

NIHSS by the lead rater (Rater 1) n (%)	NIHSS by a second independent rater (Rater 2) n (%)					
	≤ 5	6–10	11–15	16–20	> 20	Total
≤ 5	106 (58.9)	12 (6.7)	1 (0.6)	0	0	119 (66.1)
6–10	5 (2.8)	22 (12.2)	6 (3.3)	0	0	33 (18.3)
11–15	0	4 (2.2)	7 (3.9)	1 (0.6)	0	12 (6.7)
16–20	0	1 (0.6)	3 (1.7)	4 (2.2)	1 (0.6)	9 (5.0)
> 20	0	0	0	1 (0.6)	6 (3.3)	7 (3.9)
Total	111 (61.7)	39 (21.7)	17 (9.4)	6 (3.3)	7 (3.9)	180 (100.0)
Percent agreement					145/180, 80.56%	
Cohen’s Kappa statistic	Simple Kappa (95% CI)				0.64 (0.55–0.74)	
	Weighted Kappa (95% CI)				0.79 (0.72–0.86)	

**Figure 1:**
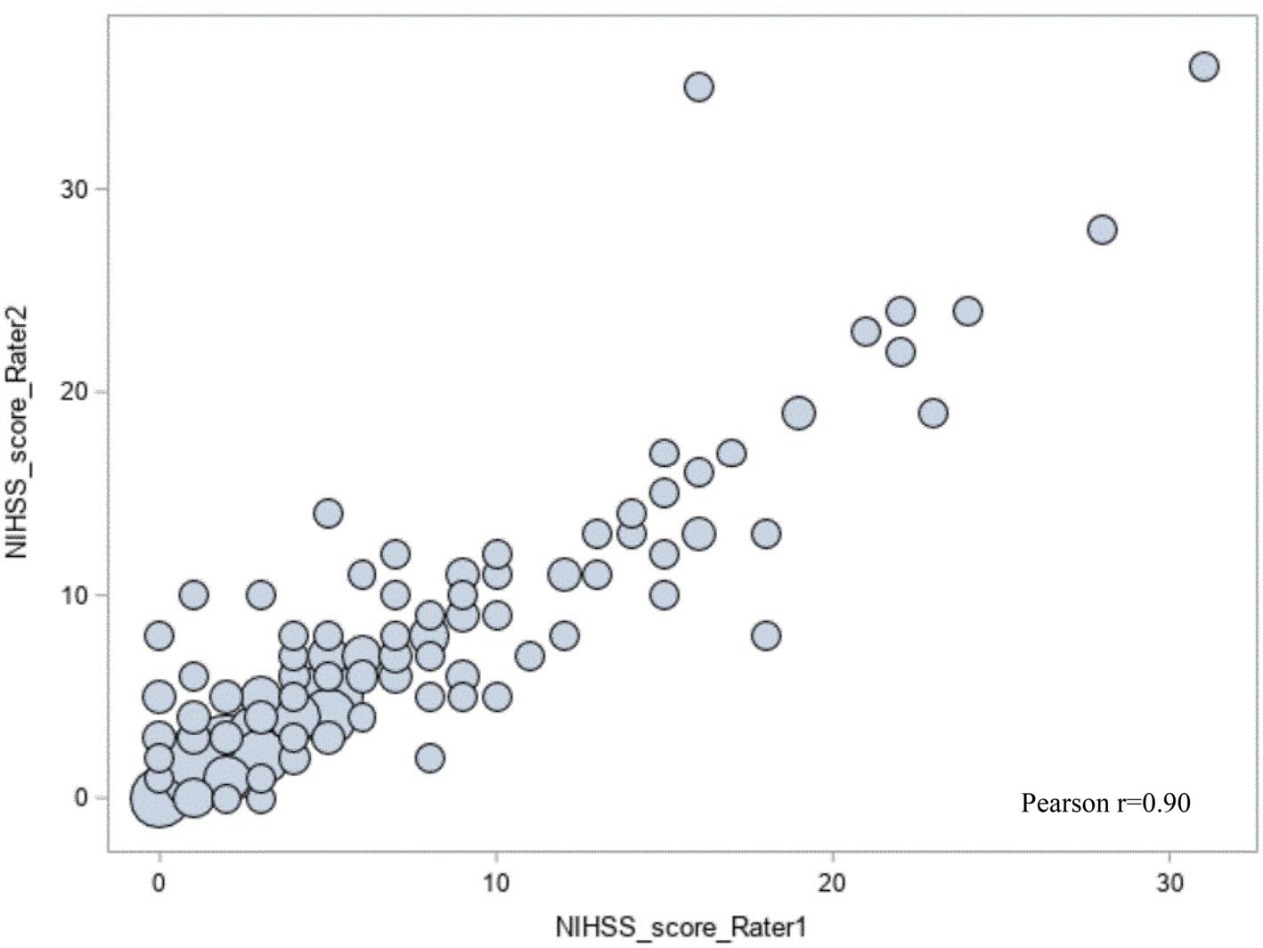
NIHSS score, interrater agreement, N = 180. Bubble size is proportional to the number of people with the specific NIHSS score.

Mean (SD) NIHSS score was 5.84 (5.88), with a median score of 4 and range 0–31 for the lead reviewer (rater 1) and mean (SD) 6.16 (6.10), median 4.5 and range 0–36 in the second independent assessment, done by one of the four secondary reviewers. Agreement between raters in NIHSS score is shown in Fig. 1. The correlation (Pearson r) between the scores reported by the lead reviewer and the second reviewers was 0.90.

## Discussion

Hospital chart-based retrospective assessment of stroke severity using the NIHSS is feasible and reliable. Using data from hospitalization charts of ischemic stroke events occurring in ARIC starting in 1987–1989, we were able to apply the retrospective algorithm developed by Williams et al. in 2000^13^ to categorize severity of ischemic stroke on admission, both in incident and recurrent events. Assessment of severity was feasible despite the variability in the extent of available data collected on stroke events occurring in ARIC over a period of 20 years.

The NIHSS is the most widely used scale for the assessment of stroke severity. It has become the “gold standard” for stroke severity rating in clinical trials^9^ on prevention, acute treatment and recovery after stroke. However, data in clinical trials and in observational studies differ; therefore, our findings contribute to the generalizability of retrospective evaluation of NIHSS. Although NIHSS is sometimes used to categorize stroke severity both in ischemic stroke and intracerebral hemorrhages, and has been reported reliable for prediction of outcome in intracerebral hemorrhages^16^, its accuracy is considered higher for evaluation of severity in ischemic compared with hemorrhagic strokes, therefore the present paper focuses on severity of ischemic stroke in ARIC.

Our findings show that the distribution of ischemic stroke severity in ARIC is skewed toward less severe strokes, with a median (25%-75%) NIHSS score of 5 (2–8). These findings are consistent with data from the 2016 National Inpatient Sample regarding NIHSS reporting in claims from the first quarter of implementation of optional reporting^17^, as well as previous findings from large clinical registries and population based studies^18,19^.

The importance of collecting NIHSS prospectively both in research and routine clinical practice is well recognized; however, our findings showing that chart-based assessment of NIHSS is feasible and reliable are useful for the majority of large epidemiologic studies on stroke that have not routinely collected data on NIHSS. The mostly qualitative information available in such studies, can be abstracted and translated into the most commonly used scale for quantitative evaluation of severity of stroke. Since severity is the most important predictor of stroke outcome, data on severity of stroke are important and useful for a wide range of studies looking at short- and long-term physical, cognitive, mental and social outcomes of stroke. The protocols for the study are available and already being adopted by a large dementia consortium.

## Conclusion

Hospital chart-based assessment of stroke severity using the NIHSS is feasible and reliable. Data on stroke severity are essential to accurately assess short- and long-term outcomes of stroke. Our findings are important and useful for a wide range of studies on stroke.

## Data Availability

The data that support the findings of this study are available from the corresponding author upon reasonable request. The protocols for the study are available and already being adopted by a large dementia consortium.

## Abbreviations

ARIC: Atherosclerosis Risk in Communities
CI: Confidence interval
NIHSS: National Institutes of Health Stroke Scale
REDCap: Research electronic data capture
SD: Standard deviation
US: United States
